# Circulating immune and biochemical markers predict tumor response to immunotherapy in advanced melanoma and lung cancer

**DOI:** 10.3389/fimmu.2026.1854351

**Published:** 2026-07-09

**Authors:** Silvia Sequero-Lopez, Diego Castillo-Barnes, Laura Gálvez-Carvajal, Natalia Palazón-Carrión, Francisco Jesús Martínez-Murcia, Luis de la Cruz-Merino, Eva Muñoz-Couselo, Mariano Provencio, Andrés Ortiz, Juan Manuel Górriz, Javier Ramírez, Antonio Rueda-Domínguez, Isabel Blancas

**Affiliations:** 1 Department of Medical Oncology, Hospital Universitario San Cecilio, Granada, Spain; 2 Faculty of Medicine, University of Málaga, Málaga, Spain; 3 IBS Granada, Instituto de Investigación Biosanitaria de Granada, Granada, Spain; 4 Communications Engineering, University of Málaga, Málaga, Spain; 5 IBIMA Plataforma Bionand, Instituto de Investigación Biomédica de Málaga, Málaga, Spain; 6 Instituto Andaluz Interuniversitario en Ciencia de Datos e Inteligencia Computacional (DaSCI), Granada, Spain; 7 Department of Medical Oncology, Hospital Universitario Virgen de la Victoria, Málaga, Spain; 8 Department of Medical Oncology, Hospital Universitario Virgen de la Macarena, Seville, Spain; 9 Signal Theory, Networking and Communications, University of Granada, Granada, Spain; 10 Centro de Investigación en Tecnologías de la Información y las Comunicaciones (CITIC-UGR), Granada, Spain; 11 Department of Medical Oncology, Hospital Universitario Vall d’Hebron, Barcelona, Spain; 12 Department of Medical Oncology, Hospital Universitario Puerta de Hierro-Majadahonda, Madrid, Spain

**Keywords:** Bayesian mixed-effects models, CD4+ T cells, circulating biomarkers, immune checkpoint inhibitors, longitudinal analysis, lymphocyte subpopulations, peripheral immune profiling

## Abstract

**Introduction:**

Peripheral immune biomarkers provide a minimally invasive approach to monitor systemic responses to cancer immunotherapy, yet their predictive value in real-world longitudinal settings remains incompletely defined.

**Methods:**

We conducted a prospective longitudinal study in patients with advanced melanoma and non-small cell lung cancer (NSCLC) receiving immune checkpoint inhibitors. Thirteen circulating immune, inflammatory, and nutritional biomarkers were analyzed across treatment cycles, and their association with radiological response over time was assessed using Bayesian ordinal mixed-effects models.

**Results:**

Nine biomarkers showed significant longitudinal associations with clinical outcomes. CD4+ T cells emerged as the strongest predictor of favorable response, followed by CD3+ and CD45+ T cells, natural killer cells, albumin, and total protein, whereas elevated neutrophils, lactate dehydrogenase, and the neutrophil-to-lymphocyte ratio were associated with poor outcomes. No significant effects were observed for CD8+ T cells, B cells, total lymphocytes, or the CD4/CD8 ratio.

**Discussion:**

These findings identify circulating CD4+ T cells as the most informative peripheral biomarker associated with longitudinal response to immune checkpoint inhibitors and support the clinical utility of peripheral immune profiling as a dynamic, minimally invasive strategy for monitoring treatment efficacy in routine oncology practice.

## 1 Introduction

Immunotherapy has transformed the therapeutic landscape of oncology by harnessing the patient’s immune system to selectively eliminate tumor cells. Unlike conventional chemotherapy, which targets rapidly dividing cells, Immune Checkpoint Inhibitors (ICIs) reactivate endogenous antitumor responses, leading to durable remissions in a subset of patients with advanced malignancies. The effectiveness of these therapies depends on the presence of functional immune cells capable of recognizing and attacking tumor antigens, both within the tumor microenvironment and systemically (1). Understanding which immune components contribute most strongly to clinical benefit remains an area of active investigation (2, 3). In particular, the characterization of circulating lymphocyte subpopulations has emerged as a promising strategy to identify peripheral biomarkers of immune competence and to predict response to immune checkpoint blockade (4–6).

In this context, circulating lymphocyte subpopulations may offer relevant prognostic information. CD8+ cytotoxic T lymphocytes play a central role in tumor cell killing, particularly when activated by antigen-presenting cells (APCs) via major histocompatibility complex class I (MHC-I) molecules (7). Their activity is further supported by CD4+ helper T cells, which not only enhance proliferation and memory formation (8), but also potentiate CD8+ T cell responses, as demonstrated by the association between circulating CD4+ Th1-like cells and clinical benefit from anti-PD-1 therapy in lung cancer patients (4). B cells can also contribute by producing antibodies against tumor-associated antigens, and their infiltration in tumors has been associated with improved prognosis (9). Natural killer (NK) cells add a layer of innate immunity, eliminating target cells without prior antigen sensitization (10). These cell types are broadly represented in the CD45+ lymphocyte compartment, measurable in peripheral blood.

However, tumor cells can evade immune surveillance by inducing functional exhaustion in lymphocytes through immunoregulatory pathways (8, 11). A central mechanism involves the expression of PD-1 (programmed cell death protein 1) on T cells, which, upon binding to its ligand PD-L1 on tumor or stromal cells, inhibits T cell receptor signaling and dampens effector functions such as cytokine production and cytotoxicity (12–14). Although PD-1 expression serves as a physiological safeguard to maintain peripheral tolerance and prevent autoimmunity, many cancers co-opt this immune checkpoint to escape immune elimination (15). This mechanism has been widely recognized as a hallmark of immune evasion in the tumor microenvironment.

Checkpoint inhibitors targeting PD-1, PD-L1, or CTLA-4 can block these suppressive interactions, reactivating lymphocytes and restoring antitumor activity (16, 17). Anti-PD-1 therapies such as pembrolizumab have dramatically improved survival in metastatic settings (18). For example, 5-year overall survival in advanced melanoma now exceeds 34–41% (19), and similar clinical benefits have been reported in lung cancer (20–23) and urothelial carcinoma (24).

Since immunotherapy is delivered systemically, its effects extend beyond the tumor site. Immune-related adverse events (irAEs) affecting multiple organs provide evidence of widespread immune activation (25, 26). This systemic response can be monitored through peripheral blood biomarkers. Several studies suggest that patients who respond to immunotherapy exhibit higher counts of circulating lymphocytes than non-responders (5, 27, 28). Additionally, systemic inflammatory markers such as the neutrophil-to-lymphocyte ratio (NLR) and lactate dehydrogenase (LDH) have been associated with poor prognosis (29, 30). Nutritional indicators, including serum albumin and total protein, are also commonly evaluated, as their decline may reflect both compromised host status and altered pharmacokinetics of IgG-based therapies, ultimately correlating with worse outcomes (7, 10, 31, 32).

Despite these advances, the longitudinal dynamics of circulating immune biomarkers and their association with treatment response in real-world clinical settings remain insufficiently characterized. In particular, the role of specific lymphocyte subpopulations as dynamic predictors of immunotherapy efficacy remains unclear. In this study, we aim to evaluate the relationship between circulating immune cell populations and radiological response to immunotherapy in patients with advanced melanoma and non-small cell lung cancer (NSCLC) using Bayesian ordinal mixed-effects models applied to prospective longitudinal data.

## 2 Materials and methods

### 2.1 Study design and population

We conducted an observational, prospective study involving patients diagnosed with NSCLC or melanoma who were receiving immunotherapy at the Medical Oncology Service of Hospital Universitario San Cecilio de Granada (HUSC). From the start of treatment, participants were prospectively monitored at each immunotherapy cycle. Peripheral blood samples were collected to quantify lymphocyte subpopulations, biochemical markers, and nutritional parameters, continuing until radiological progression or a predefined data cutoff.

Eligibility criteria included age ≥ 18 years, diagnosis of stage III or IV NSCLC or melanoma, ongoing systemic immunotherapy, availability of serial blood counts at each treatment cycle, and assessment of radiological response according to iRECIST criteria [complete response (CR), partial response (PR), stable disease (SD), progressive disease (PD)] by Computed Tomography (CT) (33).

Patients were recruited by oncologists during routine visits at the outpatient clinic. The study did not interfere with clinical decision-making or alter standard-of-care protocols.

The dataset comprises a total of 141 patients enrolled between 2019 and 2024. The median age was 65 years (range 20–83), with a predominance of males (70.92%, n = 100). NSCLC accounted for 60.28% (n = 85) of the cases, while 39.72% (n = 56) had melanoma. Most patients were diagnosed at stage IV (72.34%, n = 102). Among lung cancer patients, PD-L1 expression over 50% was observed in 29.41% (n = 25), and 25.88% (n = 22) presented relevant genetic alterations (BRAF, KRAS, MET, CDK4, FGFR3, PIK3CA, MAP2K2). In melanoma patients, BRAF V600E mutation was found in 41.07% (n = 23). At the data cutoff, 36.43% of all patients were alive, including 23.53% (n = 20) of NSCLC cases and 55.36% (n = 31) of melanoma cases.

**Figure 1:**
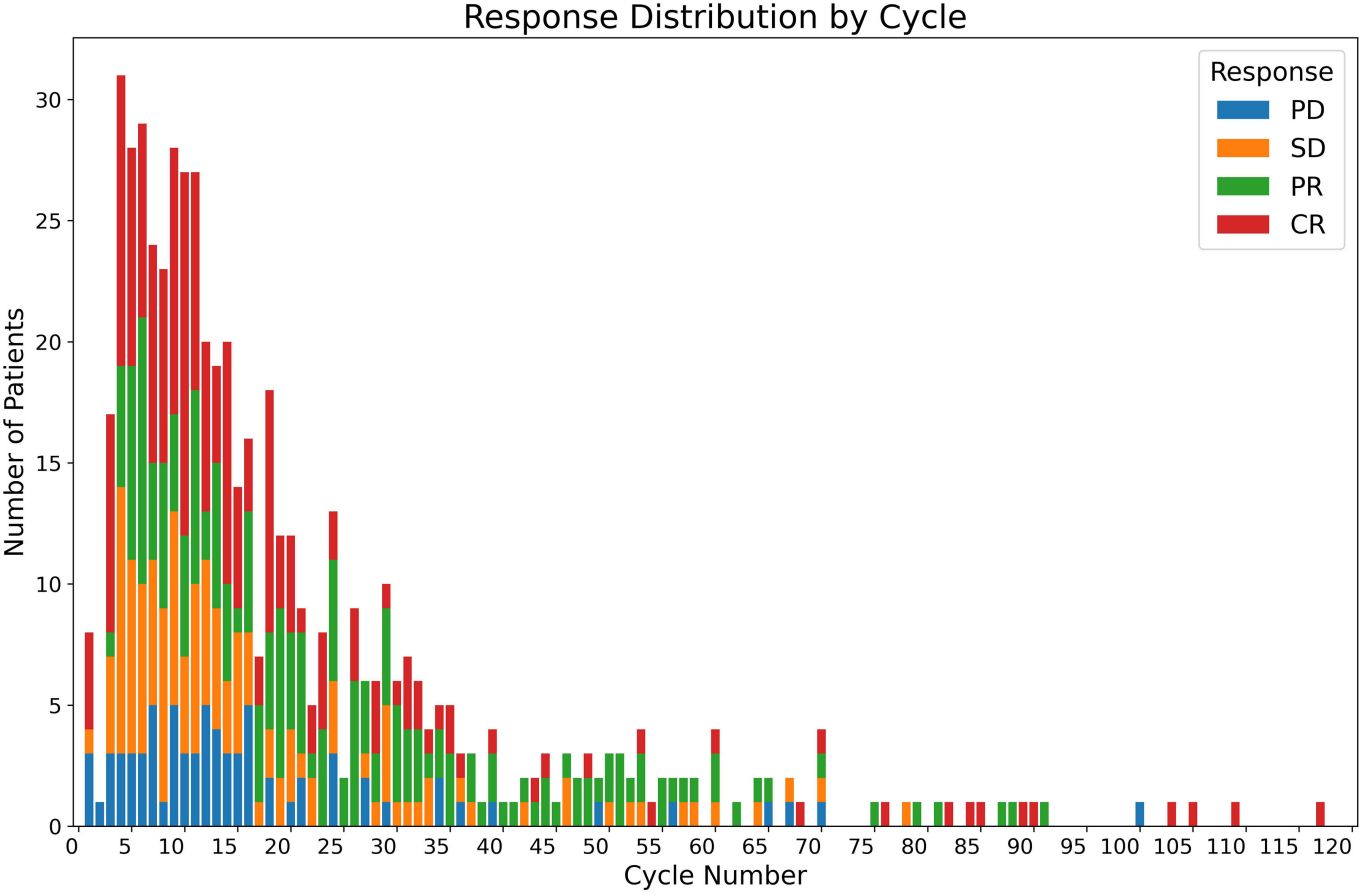
Distribution of radiological responses by immunotherapy cycle. CR, Complete Response; PR, Partial Response; SD, Stable Disease; PD, Progressive Disease.

**Figure 1** shows the number of patients receiving each immunotherapy cycle, stratified by radiological response. Most responses are observed during the first 15 cycles, with complete and partial responses (CR, PR) becoming increasingly frequent in later cycles.

The primary objective was to assess whether peripheral blood lymphocyte subpopulations differ between patients achieving clinical benefit (CR, PR, or SD) and those experiencing disease progression (PD) during immunotherapy. Secondary objectives included the evaluation of NLR, LDH, total protein, and albumin in both responders and non-responders.

### 2.2 Data collection and variables

For each patient, peripheral blood samples were collected before each immunotherapy cycle in routine clinical visits. Data collection included absolute counts of lymphocyte subpopulations (CD3+ T, CD4+ T, CD8+ T, B cells, NK cells, CD45+), as well as total neutrophils and lymphocyte counts. Lymphocyte profiling was performed using the BD FACSLyric™ Flow Cytometry System^1^, while hematological and biochemical parameters were measured using the Abbott Alinity H-Series and Abbott Alinity C-Series^2^.

Biomarkers were recorded longitudinally at each treatment cycle, up to radiological progression or data cutoff. Due to the clinical nature of follow-up, measurement intervals were not uniform across patients but followed their respective treatment schedules (e.g., cycle 1, 6, 9, etc.). Radiological response was assessed at scheduled imaging timepoints using iRECIST criteria (33).

### 2.3 Statistical analysis

To assess the relationship between immune biomarkers and treatment response, we employed Bayesian ordinal mixed-effects models. These models account for both between-subject and within-subject variability and are well suited for longitudinal clinical data with non-uniform but clinically consistent follow-up times (34).

Radiological response was defined according to iRECIST criteria and modeled as an ordinal outcome (PD < SD < PR < CR). For each biomarker, a separate ordinal regression model was fitted using treatment response as the dependent variable. Predictor variables were standardized prior to analysis. The model included a random intercept for each patient and assumed proportional odds across response levels. Inference was conducted in a fully Bayesian framework using Hamiltonian Monte Carlo sampling (35).

Model convergence was assessed using the Gelman-Rubin statistic (R-hat), effective sample size (ESS), and inspection of trace plots. Statistical significance of each biomarker was evaluated via the posterior distribution of the estimated coefficient, using 95% credible intervals (CI) and empirical two-tailed p-values derived from Monte Carlo sampling.

## 3 Results

We analyzed eleven circulating biomarkers, along with two derived ratios (NLR and CD4/CD8), using a Bayesian ordinal mixed-effects model that accounts for repeated measurements across 141 patients (total: 568 observations). Each biomarker was included as a fixed effect, while patient identity was modeled as a random intercept.

**Table 1** summarizes the coefficient estimates, 95% credible intervals, and model diagnostics. Convergence was confirmed for all models (R-hat ≈ 1.000) with effective sample sizes (ESS) above 2000.

**Table 1:** Bayesian mixed-effects model results for circulating and derived biomarkers.

Biomarker	Coef.	p-value	95% CI (low)	95% CI (up)	R-hat	ESS
CD4+ T	0.974	<0.001	0.528	1.455	1.001	2618
Total neutrophils	-0.852	<0.001	-1.225	-0.492	1.000	5631
LDH	-0.893	<0.001	-1.280	-0.520	1.002	4662
NLR	-4.615	<0.001	-6.251	-3.034	1.000	4674
CD45+	0.711	0.0003	0.295	1.118	1.000	3380
CD3+ T	0.739	0.0003	0.323	1.197	1.001	3192
Total protein	0.432	0.0075	0.120	0.738	1.000	5727
Albumin	0.475	0.0080	0.114	0.862	1.000	5372
NK	0.480	0.0100	0.114	0.846	1.001	4437
CD8+ T	0.386	0.0928	-0.069	0.829	1.001	3330
Total lymphocytes	0.213	0.1288	-0.061	0.520	1.001	7542
CD4/CD8	0.405	0.1608	-0.153	0.994	1.002	2201
B cells	0.202	0.4143	-0.293	0.698	1.000	4443

**Figure 2:**
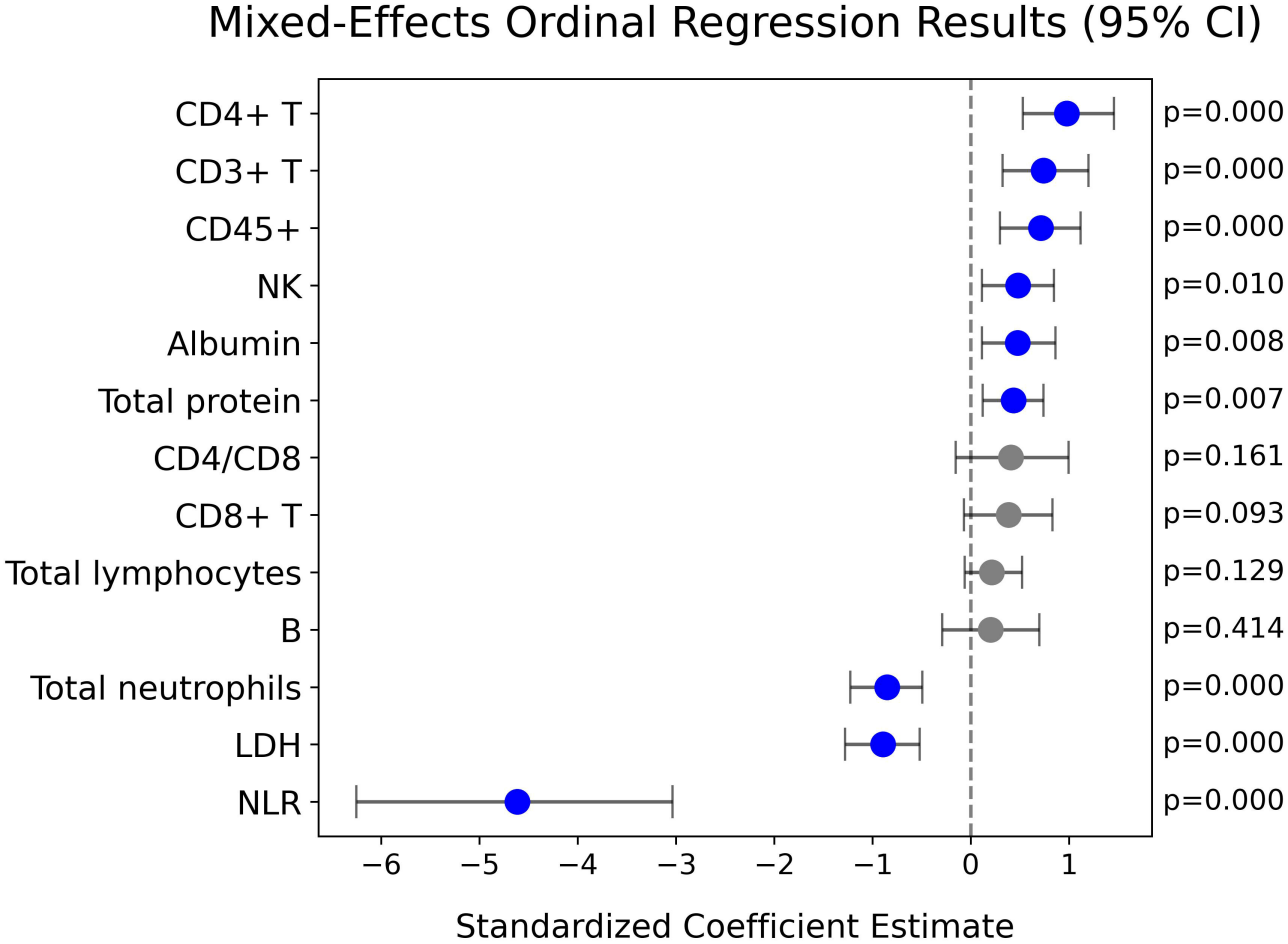
Forest plot of standardized coefficient estimates and 95% CI for each biomarker in the ordinal mixed-effects model. Blue points indicate biomarkers significantly associated with treatment response (posterior p-value < 0.05), while gray points denote non-significant effects. A vertical dashed line marks the null effect at zero.

The Forest plot shown in **Figure 2** summarizes the estimated coefficients and their 95% credible intervals. CD4+ T cells showed the strongest positive association with treatment response. Other biomarkers significantly associated with favorable outcomes included CD3+ and CD45+ T cells, NK cells, albumin, and total protein, whereas elevated neutrophils, LDH, and NLR were associated with poorer response. In contrast, CD8+ T cells, total lymphocytes, the CD4/CD8 ratio, and B cells did not reach statistical significance.

**Figure 3:**
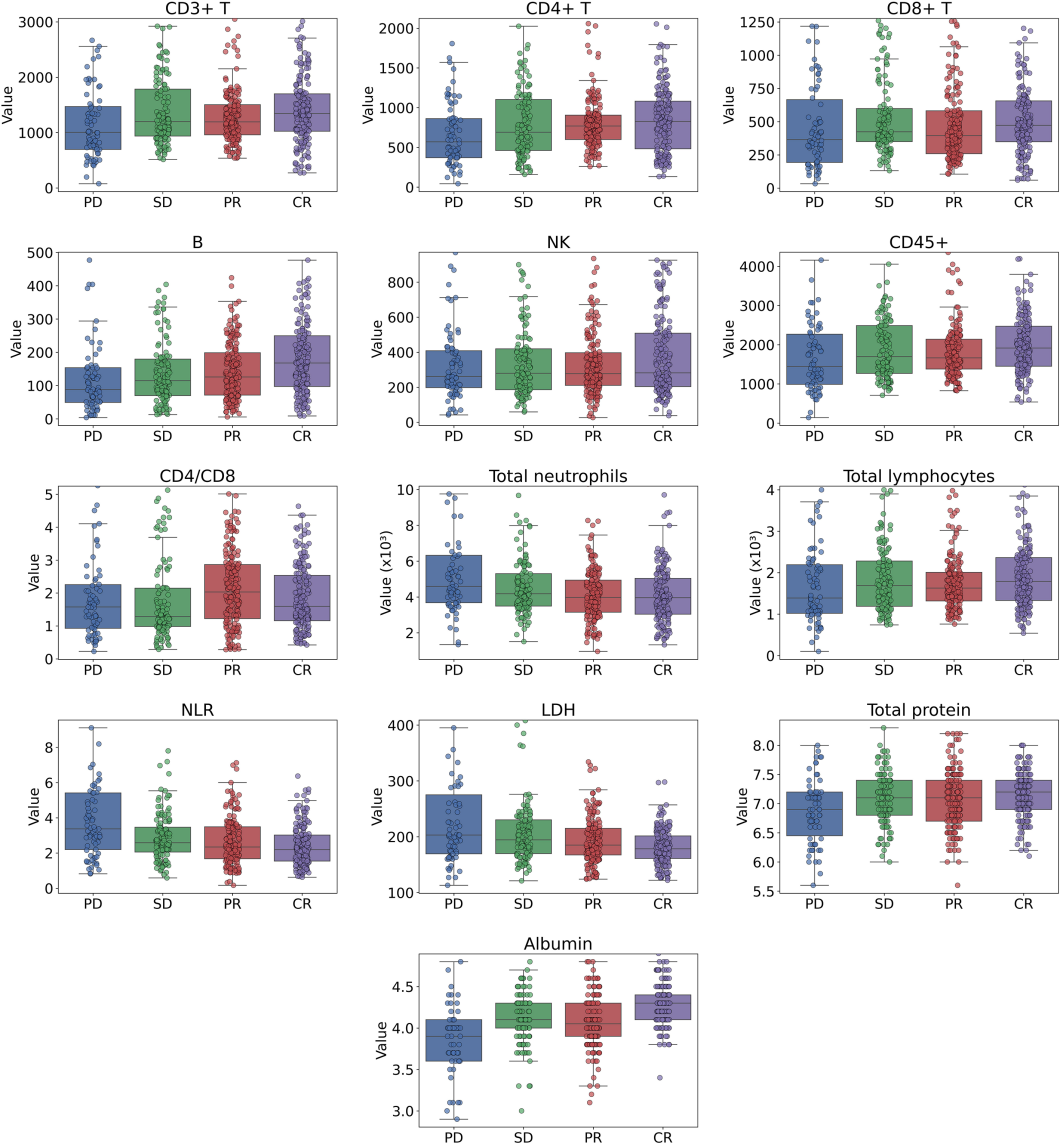
Boxplots and scatterplots of immune and biochemical biomarkers stratified by treatment response (PD, SD, PR, CR) highlighting biomarker variation across clinical response categories.

The distribution of each biomarker across clinical response groups is visualized in **Figure 3**, highlighting the stratification patterns and variability in peripheral blood markers.

Two additional exploratory analyses are provided in **Supplementary Tables 1**, **2**, focusing on early-cycle biomarker dynamics and potential responder subphenotypes among patients achieving PR or CR.

## 4 Discussion

Peripheral immune profiling offers a practical window into systemic responses to immunotherapy. In this longitudinal study, we used Bayesian mixed-effects models to identify circulating biomarkers associated with clinical benefit. Among them, CD4+ T cells emerged as the strongest predictor of favorable response.

### 4.1 Peripheral lymphocyte subsets

CD4+ T cells showed the strongest association with radiological response [posterior coefficient = 0.974, 95% credible intervals (0.528, 1.455), p < 0.001], reinforcing their role as systemic indicators of a clinically effective antitumor immune response. These cells play a central role in adaptive immunity, coordinating CD8+ and myeloid cell responses while also exerting direct cytotoxicity via MHC class II-restricted killing and producing effector cytokines (1, 36, 37). Their activity involves IL-2-driven clonal expansion and the secretion of interferon-γ (IFNγ), and tumor necrosis factor-*α* (TNF*α*).

Importantly, systemic CD4+ T responses often target self-derived epitopes, enabling immune activation even in tumors with low neoantigen loads (1, 14). Our findings align with previous studies showing that high peripheral levels of effector CD4+ T cells predict durable responses to checkpoint blockade in NSCLC and melanoma (4, 38, 39). These subsets correlate with PD-1+ CD8+ T-cell expansion and long-term survival, suggesting a crucial role in sustaining antitumor immunity (4). Consistent with this concept, exploratory subgroup analyses further suggested a tendency toward higher CD4+ T-cell levels among CR compared with PR (**Supplementary Table 2**), a pattern that was less evident for CD3+ and CD8+ T-cell populations. Moreover, CD4+ T cells actively reshape the tumor microenvironment by reprogramming tumor-associated myeloid cells toward pro-inflammatory phenotypes, thereby overcoming local immunosuppression (11, 40, 41). This immunological remodeling capability, coupled with their accessibility via peripheral blood, positions circulating CD4+ T cells as integrative biomarkers of both systemic immune activation and local immune engagement (42).

CD3+ and CD45+ cells were also significantly associated with favorable outcomes [CD3+: posterior coefficient = 0.739, 95% credible intervals (0.323, 1.197); CD45+: posterior coefficient = 0.711, 95% credible intervals (0.295, 1.118); p < 0.001 for both], supporting their relevance as systemic indicators of immune competence and activation. CD3+ T cells reflect the total T-cell compartment and have been linked to general immune fitness and responsiveness to checkpoint inhibitors (9), while CD45+ cells, encompassing globally activated leukocytes, reflect the capacity to initiate effector responses. Although less specific than subsets such as CD4+ or CD8+, these markers may capture the immune system’s baseline readiness to mount coordinated antitumor responses (27, 37), and their broad scope and accessibility make them potential components of composite biomarkers for patient stratification.

NK cells were significantly associated with improved radiological response [posterior coefficient = 0.480, 95% credible intervals (0.114, 0.846), p = 0.010], highlighting the relevance of innate immunity in antitumor surveillance. As cytotoxic lymphocytes that act independently of prior antigen exposure, NK cells provide a rapid and nonspecific immune defense, complementing adaptive T-cell–mediated mechanisms (37, 41, 43, 44). Their ability to eliminate tumor cells through direct cytotoxicity, as well as to modulate dendritic cells and macrophages via cytokine release, contributes to shaping the immune contexture of tumors (10). Notably, their activity becomes especially relevant in tumors with low MHC class I expression, where CD8+ T-cell recognition is impaired (10). In this sense, elevated peripheral NK cell levels may serve as compensatory indicators of innate-driven immune readiness, particularly in patients with limited adaptive activation. From a translational perspective, NK cells may enhance the predictive accuracy of composite biomarkers, providing added value in stratifying patients who may benefit from immunotherapy even in the absence of robust T-cell priming (45).

Peripheral CD8+ T cells, the CD4/CD8 ratio, and B lymphocytes did not show statistically significant associations with response (CD8+ T cells: p = 0.093; CD4/CD8 ratio: p = 0.161; B cells: p = 0.414). Although CD8+ T cells displayed a trend toward a positive association, the high interindividual variability limits the robustness of this finding. This is consistent with the notion that key CD8+ T cell activity may predominantly occur at the tumor site rather than in peripheral circulation. Several studies have highlighted that tumor-infiltrating CD8+ T cells exhibit dynamic, clonally expanded, and often exhausted phenotypes associated with local tumor control, suggesting that peripheral blood assessments may fail to capture their functional relevance (14, 46–48).

Similarly, the CD4/CD8 ratio, often considered a surrogate marker of immune equilibrium, did not reach statistical significance. While conceptually useful, this ratio may obscure the specific and non-redundant roles of CD4+ and CD8+ subsets, whose individual activation and exhaustion states may be more informative than their relative abundance (4). B cell levels were also not significantly associated with response, in line with previous findings pointing to a more limited role for circulating B cells in checkpoint blockade (7, 36, 49). It is plausible that their contribution is more pronounced at the tissue level, particularly within tertiary lymphoid structures, where they may support antigen presentation and intratumoral immune crosstalk (11).

### 4.2 Inflammatory and systemic biomarkers

Neutrophil and lymphocyte counts, as well as their derived ratio (NLR), were informative markers of systemic inflammation and immune suppression in our cohort. These variables are established markers of systemic inflammation and immune suppression (50, 51). In our analysis, neutrophil count was negatively associated with radiological response [posterior coefficient = -0.852, 95% credible intervals (-1.225, -0.492), p < 0.001], consistent with their role in promoting tumor progression and inhibiting cytotoxic immunity (52). NLR showed the strongest negative effect among all biomarkers [posterior coefficient = -4.615, 95% credible intervals (-6.251, -3.034), p < 0.001], underscoring its prognostic relevance. Elevated NLR values, typically >3–5, reflect a dominance of protumoral innate responses over adaptive surveillance, with reductions in CD4+ and CD8+ T cells and increases in immunoregulatory populations (30), findings that are also supported by recent longitudinal analyses of dynamic inflammatory biomarkers during immunotherapy (55). In contrast, lymphocyte count alone showed a non-significant trend toward better outcomes (posterior coefficient = 0.213, 95% credible intervals [-0.061, 0.520], p = 0.129), possibly due to the functional heterogeneity of circulating lymphocyte subsets.

LDH, albumin, and total protein further highlighted the relevance of systemic host condition in treatment response. LDH, a surrogate of tumor burden and glycolytic metabolism, was negatively associated with radiological response [posterior coefficient = -0.893, 95% credible intervals (-1.280, -0.520), p < 0.001]. Its elevation, linked to hypoxia, acidosis and immune dysfunction, has been implicated in immunotherapy resistance across NSCLC and melanoma (17, 19, 20, 23, 52), alongside other emerging peripheral inflammatory biomarkers such as serum ferritin (56). In contrast, nutritional markers such as albumin and total serum protein were positively associated with better outcomes. Albumin, a negative acute-phase reactant and proxy of nutritional and inflammatory status, showed a favorable association with response [posterior coefficient = 0.475, 95% credible intervals (0.114, 0.862), p = 0.008], as did total protein [posterior coefficient = 0.432, 95% credible intervals (0.120, 0.738), p = 0.008]. These findings are consistent with previous reports linking albumin levels > 4.0–4.5 g/dL to longer survival and better immunotherapy tolerance in lung and colorectal cancer (5, 6, 31, 32). Beyond their prognostic value, recent studies suggest these markers also reflect immune competence, being associated with increased CD4+ and NK cell activity (51, 53), a pattern also observed in our cohort.

Two additional exploratory analyses are provided in **Supplementary Tables 1**, **2**, focusing on early-cycle biomarker dynamics and potential responder subphenotypes among patients achieving PR or CR.

### 4.3 Interpretation and clinical implications

These findings support the use of peripheral immune profiling as a dynamic and minimally invasive approach to monitor treatment efficacy in cancer immunotherapy. By applying Bayesian mixed-effects models to real-world longitudinal data, we identified specific lymphocyte subsets (most notably CD4+ T cells) as strong correlates of clinical response. This analytical framework may help inform future strategies for risk stratification and personalized treatment (54), highlighting the prognostic relevance of circulating immune cells as accessible biomarkers in routine care.

Despite these findings, some limitations should be considered. The analysis was restricted to peripheral blood biomarkers and therefore does not capture tumor-infiltrating immune populations, which may provide complementary information on local immune dynamics. Variability in treatment schedules and follow-up intervals, inherent to real-world longitudinal data, may also introduce heterogeneity that is not fully accounted for by the model. Although exploratory analyses were performed for early treatment dynamics (**Supplementary Table 1**), the study was not specifically powered to evaluate primary resistance during the first immunotherapy cycles or to perform robust subgroup analyses according to tumor type, metastatic site distribution, PD-L1 expression status, or specific molecular tumor characteristics. As a consequence, potential biological differences between NSCLC and melanoma patients, as well as between individual molecular subgroups, could not be reliably assessed within the current dataset. External validation in independent cohorts would further strengthen the robustness and generalizability of these findings.

Future studies should aim to further characterize the dynamic interplay between circulating immune biomarkers and treatment response, and to explore how longitudinal monitoring strategies can be integrated into clinical decision-making to improve patient stratification and treatment optimization.

## 5 Conclusions

This prospective study evaluated a panel of peripheral immune and biochemical biomarkers in patients with advanced lung cancer or melanoma receiving immune checkpoint inhibitors, using Bayesian ordinal mixed-effects models tailored for real-world longitudinal data. Nine biomarkers were significantly associated with radiological response.

CD4+ T cells emerged as the most robust predictor of clinical benefit, reinforcing their central role in antitumor immunity. Additional markers associated with favorable outcomes included CD3+ and CD45+ T cells, NK cells, and host-related indicators such as albumin and total protein, whereas elevated neutrophils, LDH, and the neutrophil-to-lymphocyte ratio were negatively associated with response. In contrast, CD8+ T cells, B cells, total lymphocytes, and the CD4/CD8 ratio did not show significant associations, highlighting the limitations of peripheral measurements for certain immune subsets.

Taken together, these findings support the clinical relevance of peripheral immune profiling as a dynamic and non-invasive approach for treatment monitoring and response prediction in cancer immunotherapy. The integration of lymphocyte subpopulation analysis into clinical workflows may improve patient stratification and support more informed therapeutic decisions. Further validation in independent cohorts and complementary integration with tumor-level immune data will help to consolidate the translational utility of these biomarkers.

## Data Availability

The datasets generated and/or analyzed during the current study are not publicly available due to ethical and legal restrictions related to patient confidentiality and data governance policies of the Hospital Universitario San Cecilio (Granada, Spain). Access to the data may be granted to qualified researchers upon reasonable request and subject to approval by the corresponding Ethics Committee. Requests should be directed to the Clinical Research Coordination Unit at investigacion.husc.sspa@juntadeandalucia.es, including a brief research proposal and evidence of ethical approval from the requesting institution.

## Footnotes

1 For more information: https://www.bdbiosciences.com/en-us/products/instruments/flow-cytometers/clinical-cell-analyzers/facslyric.

2 For more information: https://www.corelaboratory.abbott/int/en/offerings/brands/alinity/alinity-ci-series.html.
