# Supplementary material for: Circulating immune and biochemical markers predict tumor response to immunotherapy in advanced melanoma and lung cancer
Source: Front Immunol. 2026 Jul 9;17:1854351. doi: 10.3389/fimmu.2026.1854351 (PMC13391877; doi:10.3389/fimmu.2026.1854351)
Supplement: Supplementary file 1 [file Table1.pdf]

Circulating Immune and Biochemical Markers Predict Tumor Response to Immunotherapy in Advanced Melanoma and Lung Cancer

Supplementary Materials

Supplementary Table S1 presents an exploratory early-cycle analysis of peripheral immune and biochemical biomarkers according to treatment response at cycle 3 or 4. A total of 41 patients had available biomarker measurements at cycle 3 or 4, including 5 patients with progressive disease (PD) and 36 patients with stable disease (SD), partial response (PR), or complete response (CR). This exploratory analysis was performed to evaluate potential early biomarker patterns associated with primary resistance to immune checkpoint inhibitors.

Biomarker	Median [IQR] (Early PD)	Median [IQR] (SD/PR/CR)	Mann-Whitney U p-value
CD4+ T	498 [390-643]	690 [409–960]	0.349
CD3+ T	760 [729-1288]	1159 [866–1493]	0.310
CD8+T	482 [220–630]	440 [277–598]	0.765
NK	200 [158–203]	373 [218–560]	0.027
CD45+	1117 [835–1934]	1641 [1285–2362]	0.189
Total neutrophils	3530 [3250–3670]	3930 [3265–5008]	0.449
Total lymphocytes	1020 [990–1800]	1560 [1233–2363]	0.182
NLR	3.19 [3.00–3.57]	2.48 [1.74–3.20]	0.320
LDH	194 [178–251]	193 [176–231]	1.000
Total protein	6.55 [6.03–7.05]	7.10 [6.80–7.40]	0.167
Albumin	3.70 [3.35–3.93]	4.20 [3.90–4.40]	0.028

Table S1. Exploratory early-cycle analysis of peripheral biomarkers according to treatment response at cycle 3 or 4. Values are reported as median [IQR]. P-values were calculated using the Mann–Whitney U test.

Supplementary Table S2 presents an exploratory responder phenotype analysis restricted to patients achieving partial response (PR) or complete response (CR). A total of 77 patients were included, comprising 37 PR patients and 40 CR patients. Patient-level median CD4+, CD3+, and CD8+ T-cell values during PR/CR observations were compared between PR and CR, and very high values were defined as those above the 75th percentile within the PR/CR subgroup.

Biomarker	Median [IQR] (PR)	Median [IQR] (CR)	p-value PR vs CR	Q75 threshold	CR among >Q75	CR among ≤Q75	Fisher p-value
CD4+ T	770 [604–878]	813 [417–1098]	0.803	961	13/19 (68.4%)	27/58 (46.6%)	0.118
CD3+ T	1215 [1028–1407]	1326 [967–1627]	0.737	1554	12/19 (63.2%)	28/58 (48.3%)	0.299
CD8+ T	420 [328–588]	448 [346–679]	0.661	674	10/19 (52.6%)	30/58 (51.7%)	1.000

Table S2. Exploratory responder phenotype analysis among patients achieving PR or CR. Patient-level biomarker values were summarized as the median across PR/CR observations. Very high values were defined as >Q75 within the PR/CR subgroup. P-values were calculated using the Mann–Whitney U test for PR versus CR comparisons and Fisher's exact test for CR enrichment above versus below Q75.
